# A Comprehensive Study on the Effect of Plasticizers on the Characteristics of Polymer Inclusion Membranes (PIMs): Exploring Butyl Stearate as a Promising Alternative

**DOI:** 10.3390/membranes14010019

**Published:** 2024-01-09

**Authors:** Berta Alcalde, Gemma Elias, Spas D. Kolev, José Alberto Méndez, Sergi Díez, Helena Oliver-Ortega, Enriqueta Anticó, Clàudia Fontàs

**Affiliations:** 1 Chemistry Department, University of Girona, C/Maria Aurèlia Capmany, 69, 17003 Girona, Spain; berta.alcalde.96@gmail.com (B.A.); gemmaelias610@gmail.com (G.E.); enriqueta.antico@udg.edu (E.A.); 2 School of Chemistry, The University of Melbourne, Melbourne, VIC 3010, Australia; s.kolev@unimelb.edu.au; 3 Department of Chemical Engineering, The University of Melbourne, Melbourne, VIC 3010, Australia; 4 Faculty of Chemistry and Pharmacy, Sofia University “St. Kl. Ohridski”, 1 James Bourchier Blvd., 1164 Sofia, Bulgaria; 5 Chemical Engineering Department, University of Girona, Edifici PI, 17003 Girona, Spain; jalberto.mendez@udg.edu; 6 Environmental Chemistry Department, Institute of Environmental Assessment and Water Research (IDAEA-CSIC), C/Jordi Girona, 18-26, 08034 Barcelona, Spain; sdsqam@cid.csic.es; 7 Department of Materials Science and Engineering, Universitat Politècnica de Catalunya, C/Colom 1, 08222 Terrassa, Spain; helena.oliver@upc.edu; 8 Institut d’Investigació Tèxtil i Cooperació Industrial de Terrassa (INTEXTER), C/Colom 15, 08222 Terrassa, Spain

**Keywords:** polymer inclusion membrane (PIM), plasticizer, butyl stearate (BTS), membrane characterization, Hg(II)

## Abstract

This study investigated the influence of various plasticizers commonly used in the manufacture of polymer inclusion membranes (PIMs), such as 2-nitrophenyl octyl ether (NPOE), phthalates, adipates, and sebacates on the mechanical, thermal, and transport properties of membranes. Additionally, butyl stearate (BTS), chosen for its non-toxic nature compared to phthalates and its cost-effectiveness relative to adipates and sebacates, was evaluated as a plasticizer in PIMs for the first time. All plasticizers were incorporated in PIMs made of either cellulose triacetate (CTA) or poly(vinyl chloride) (PVC) as the base polymers and the task-specific ionic liquid trioctylmethylammonium thiosalicylate (TOMATS) as the carrier. The plasticizers were found to significantly affect the characteristics of membrane hydrophilicity, mechanical flexibility, and thermal stability. Transport experiments using Hg(II) as a model target ion revealed that, for CTA-based PIMs, the plasticizer did not significantly affect transport efficiency. However, for PVC-based PIMs, BTS exhibited better efficiency when compared to NPOE. These findings highlight the potential of BTS as an attractive alternative to currently used plasticizers in PVC-based PIM formulations.

## 1. Introduction

Polymer inclusion membranes (PIMs) are polymeric membranes containing specific extractants and designed for the selective separation of various chemical species. Their polymeric matrix serves as their structural backbone, providing mechanical stability to the membrane, while their extractant is responsible for interacting with target chemical species [1]. Other components, such as plasticizers, can be added to the formulation of a PIM to improve both its mechanical and transport characteristics. Understanding and studying the interaction between a polymer matrix and an organic phase (extractant and plasticizer) is necessary for gaining deeper insights and knowledge into how these PIM components affect the mechanical and physical properties of membranes.

Different polymers can be used for PIMs but the most extensively studied polymers are cellulose triacetate (CTA) and poly(vinyl chloride) (PVC). Both CTA and PVC are thermoplastic polymers consisting of linear polymer chains with no cross-linking between them that can be easily dissolved in suitable organic solvents for casting PIMs.

The choice of PIM extractant (often called carrier) is crucial for membrane interactions with chemical species to be extracted. Carriers are most often acidic, basic, neutral, or solvating in nature [1]. Recently, task-specific ionic liquids (TSILs) have gained prominence as versatile extractants [2]. These ionic liquids are tailor-made for specific applications, providing unique properties that make them ideal for selective chemical interactions.

Plasticizers play a crucial role in enhancing the flexibility and workability of polymers, and historically, phthalates have been the most widely used class of plasticizers [3]. Phthalates, such as diethylhexyl phthalate (DEHP) and dibutyl phthalate (DBP), have been extensively employed in various industries for decades [4]. However, due to growing concerns over their potential health and environmental impacts, alternative plasticizers have gained attention. Adipates and sebacates are among the notable alternatives explored. Adipates, like dioctyl adipate (DOA), and sebacates, such as dioctyl sebacate (DOS), have been investigated for their plasticizing properties and are considered as more environmentally friendly options [5]. However, given their high costs, exploring new reagents as substitutes is worth considering. Butyl stearate (BTS) is a synthetic, oil-based ester that can be used as a plasticizer and lubricant. It is commonly employed as an emollient agent in personal care products, as a plasticizer in nail polishes, and as a masking agent in perfumes. Moreover, it is applied in paints and coatings and in the food industry as a flavouring and emulsifying agent [6].

In the specific context of PIMs, it is widely acknowledged that, beyond its primary function of imparting flexibility, a plasticizer can act as a solvating medium for carriers, establishing continuous pathways between the two interfaces of the membrane [7], and, therefore, facilitating the transport of the target chemical species [8,9]. Typical plasticizers utilized in the production of PIMs include derivatives of the previously mentioned compounds. Additionally, the specific reagent 2-nitrophenyl octyl ether (NPOE) is extensively used as a plasticizer in both CTA- and PVC-based PIMs [10]. This reagent possesses a high dielectric constant and low viscosity—parameters often associated with the transport efficiency of PIMs. It is widely acknowledged that the inclusion of a high-viscosity plasticizer can hinder PIM diffusion, consequently leading to a reduction in membrane transport efficiency [11]. The dielectric constant is expected to influence the dissociation of the adduct formed between the extracted chemical species and the carrier, which, coupled with the size and lipophilicity of these two species, may determine the transmembrane transport mechanism [12]. The effect of a plasticizer’s nature on PIM performance has extensively been investigated in the literature. A study conducted by Qiu et al. [13] investigated the recovery of Cu(II) using PVC-based PIMs. Four different 2-aminomethylpyridine derivatives were used as carriers as well as three plasticizers, i.e., NPOE, one phthalate, and one adipate. The PIMs were characterized by Fourier-transform infrared spectroscopy (FT-IR), X-ray photoelectron spectroscopy (XPS), and small-angle X-ray scattering (SAXS). The results of this study demonstrated that NPOE was more efficient than the other two plasticizers tested. This result was attributed to the higher dielectric constant of NPOE, which could enhance the mobility of the Cu(II) adducts formed in the membrane. Eyupoglu et al. [14] studied the extraction and removal of Cd(II) using PVC-based PIMs containing symmetrical room-temperature ionic liquids as carriers and several plasticizers. The results showed that the PIMs containing ethers as plasticizers (e.g., NPOE and 2-nitrophenyl phenyl ether (NPPE)) had a higher extraction efficiency compared to those containing adipate and phosphate plasticizers. However, the difference in Cd(II) transport of the PIMs with the different plasticizers was found to be minor. The concentration of a plasticizer in a PIM is also an important factor for membrane permeability. San Miguel et al. [15] investigated PIM permeability for In(III) as a function of the amount of bis(2,4,4-trimethylpentyl)phosphinic acid (Cyanex 272) as a carrier in the cases of NPOE and TBEP as plasticizers. Permeability values were found to increase upon the increase in the carrier content in a similar manner for both NPOE and TBEP.

In a previous study, a PIM made of CTA and the TSIL trioctylmethylammonium thiosalicylate (TOMATS) as a carrier was investigated for the transport of Hg(II) from natural water to a receiving phase consisting of 10^−3^ M cysteine [16]. In this study, it was found that the addition of the plasticizer NPOE was necessary to improve transport, even though TSIL exhibited plasticizing properties. A PIM made of 70% CTA and 30% TOMATS only transported 4% of Hg(II) in 24 h. However, after adding 20% NPOE to the PIM composition (i.e., 50% CTA, 30% TOMATS, 20% NPOE), transport increased up to 84%.

The aim of this study was to assess the effect of a plasticizer on the physical and mechanical characteristics of PVC- and CTA-based PIMs and on their transport performance, employing Hg(II) as a model target chemical species. In addition to the conventional plasticizers usually used in PIM production, the potential of BTS as a plasticizer for PIMs was also explored with the objective of finding a less toxic and expensive alternative to conventional plasticizers such as DEHP and adipate and sebacate compounds. To the best of our knowledge, BTS has not been used as a plasticizer in PIMs, and its potential use as PIM plasticizer could contribute to the broader industrial application of PIMs.

## 2. Materials and Methods

### 2.1. Reagents and Solutions

The polymers CTA (Fluka, Buchs, Switzerland) and PVC (Sigma-Aldrich, St. Louis, MI, USA) were used for the PIM preparation. CHCl_3_ stabilized with ethanol was used to dissolve CTA, whereas tetrahydrofuran (THF) was used as the solvent in the preparation of the PVC-based PIMs. Both organic solvents were acquired from Panreac (Castellar del Vallès, Spain). The TSIL trioctylmethylammonium thiosalicylate (TOMATS), used as the PIM carrier, was prepared as described by Elias et al. [17] from the commercial reagent trioctylmethylammonium chloride (Aliquat 336; Sigma-Aldrich, St. Louis, MI, USA) and sodium thiosalicylate (TCI, Tokyo, Japan). All the plasticizers used in this study were of a Selectophore grade, except BTS, which was of a technical grade. Moreover, besides the suppliers for each reagent, their prices are included in parentheses to account for the cost of a PIM as a factor to be considered. These prices are for Spain in November 2023. NPOE (EUR 8.24/mL), diethylhexyl phthalate (DEHP) (EUR 14.6/mL), 40–60% BTS (EUR 0.014/mL), and bis(1-butylpentyl) adipate (BBPA) (EUR 22/mL) were purchased from Sigma-Aldrich (St. Louis, MI, USA), and di(2-ethylhexyl) sebacate (DOS) (EUR 15/mL) and dibutyl sebacate (DBS) (EUR 25/mL), from Fluka (Buchs, Switzerland). The main characteristics and chemical structures of these compounds are shown in Table 1.

Hg(II) working solutions were prepared by the appropriate dilution of a Hg(II) ICP standard of 1000 mg L^−1^ ± 2 mg L^−1^ in HNO_3_ (12% *w*/*w*) (Fluka, Buchs, Switzerland). The feed phase consisted of simulated natural water (SNW) containing 0.5 mg L^−1^ Hg(II), 2.0 mM NaHCO_3_ (Panreac, Castellar del Vallès, Spain), 1.0 mM CaCl_2_·H_2_O (Alco-Chem, Canton, OH, USA), and 0.5 mM Na_2_SO_4_ (Merck, Darmstadt, Germany). A 10^−3^ M solution of L-cysteine (Cys) purchased from Merck (Darmstadt, Germany) was used as the receiving phase, prepared daily.

If not indicated otherwise, all other reagents and solvents were of an analytical grade. All solutions were prepared in ultrapure water (Milli-Q Plus; Millipore Ibérica S.A., Barcelona, Spain).

### 2.2. PIM Preparation

PIMs were prepared by the solvent evaporative casting method described elsewhere [16]. The PIM composition was 50% polymer (CTA or PVC), 30% TOMATS, and 20% plasticizer, as this composition was found to be effective in the transport of Hg(II) [16]. All PIM compositions are quoted in mass percentages. Moreover, membranes without plasticizer (50% polymer and 50% TOMATS) as well as films of the polymers CTA and PVC (100%) were also prepared for comparison purposes.

### 2.3. Characterization of PIMs

#### 2.3.1. Contact Angle Measurements

The contact angles of the membranes’ surfaces were measured using a commercial contact angle device, the Drop Shape Analyzer DSSA25, equipped with a video system (Krüss, Hamburg, Germany) and controlled using Krüss Advance software (version 1.3.0.0). The contact angle was measured by dropping 5 µL of ultrapure water on the surface of the membrane through a needle attached to the instrument and determining the mean value of the contact angle over a period of 60 s.

#### 2.3.2. Scanning Electron Microscopy (SEM) Imaging

The scanning electron microscopy (SEM) images of the membranes were obtained with a field emission scanning electron microscope (FE-SEM) (Model S-4100; Hitachi, Tokyo, Japan) after placing the PIM samples on stubs and coating them with carbon (Model K950 turbo evaporator; Emitech, Montigny-le-Bretonneux, France). The image processing software Quartz PCI program (Vancouver, BC, Canada) was used to collect and process the obtained images.

#### 2.3.3. Mechanical Analysis

The mechanical properties of the membranes were measured using a universal testing machine (Hounsfield Instron 2.5; Instron, Spain) with the software Data Hawk (Instron, Spain). The samples used in these tests were 0.5 cm in width and 4.0 cm in length (the thickness of the CTA-based PIMs was 55 µm, while the PVC-based PIMs were 145 µm), and before testing them, they were stored at 23 °C and 50% relative humidity for 24 h. From these tests, the values of maximum deformation, ultimate tensile strength (UTS), and elastic modulus were obtained.

#### 2.3.4. Thermogravimetric Analysis

The thermal properties of the PIMs were studied by thermogravimetric (TGA) and differential thermogravimetric (dTGA) analysis using a Mettler Toledo TGA/DSC combined instrument (Mettler Toledo, l’Hospitalet de Llobregat, Spain). A comparative study of the pure components of the membrane (polymer, carrier, and plasticizer) as well as the corresponding membrane was carried out. Each analysis was performed using approximately 10 mg of the samples in a temperature range from 30 to 650 °C, at a heating rate of 10 °C min^−1^, under a nitrogen atmosphere (40 mL min^−1^).

### 2.4. Hg(II) Transport Experiments

Transport experiments were performed according to the procedure described by Elias et al. [16], using a device with a membrane area of 1.8 cm^2^ contacting the feed solution (100 mL 0.5 mg L^−1^ Hg(II) in SNW) from one side and the receiving solution from the other side (5 mL 10^−3^ M L-cysteine). For this study, membranes of similar thickness (55 µm) for both CTA and PVC membranes were used. The feed solution was continuously stirred on a magnetic stirrer (Multistir KS 260 Basic; IKA, Staufen, Germany). Transport experiments were conducted over a 24 h period, with the exception of the kinetic studies, where different PIM devices were stopped at various times. The concentration of Hg(II) in both the feed and stripping solutions was measured using an inductively coupled plasma optical emission spectrometer (ICP-OES) (Agilent 5100 Vertical Dual View ICP-OES; Agilent Technologies, Tokyo, Japan). The membrane system’s efficiency was assessed in terms of transport efficiency (TE), calculated using the following equation:

(1) $$TE\;(\%)=(\frac{V_{r}}{V_{s}})\times (\frac{{\left[M\right]}_{r,t}}{{\left[M\right]}_{f,i}})\times 100$$

where *V_r_* is the volume of the receiving solution, *V_f_* is the volume of the feed solution, [*M*]*_r,t_* denotes the metal concentration in the receiving solution at time *t*, and [*M*]*_f,i_* is the initial metal concentration in the feed solution.

## 3. Results

### 3.1. Characterization of PIMs

Understanding and studying the interaction between the polymer matrix and the organic liquid phase (extractant and plasticizer) is crucial for gaining insights into how these components interact and influence the mechanical and physical properties of a membrane. Therefore, different techniques were used for the characterization of PIMs of different plasticizer compositions.

#### 3.1.1. Contact Angle Measurements

Contact angle measurements were conducted for each membrane to assess their wettability, which is an important factor that can affect the performance of PIMs. Kunene et al. [20] stated that hydrophilic PIMs exhibited poorer stability compared to hydrophobic ones, due to carrier leaching. However, hydrophobic PIMs generally demonstrated lower extraction efficiencies than their hydrophilic counterparts. Therefore, it is important to achieve an acceptable compromise between membrane stability and extraction efficiency. 

The hydrophilic nature of all studied PIMs (Table 2) was evident, as their contact angles were below 90° [21]. The introduction of both the extractant and the extractant + plasticizer enhanced the hydrophilic characteristics of the PIMs. This effect can be attributed to the presence of TOMATS, an ionic compound, and the plasticizer, in the PIM matrix. This effect is consistent with the findings of other researchers [22,23,24].

Specifically, the contact angle of the pure CTA membrane decreased from 62.62° to 49.27° upon the addition of 50% TOMATS in the PIM composition. When comparing the membranes with plasticizers, with the exception of DOS, all other plasticizers had a pronounced impact of increasing the hydrophilic character of the corresponding PIMs compared to the membrane containing only the polymer and TOMATS, with the plasticizers BTS and BBPA exhibiting the most significant effects.

Concerning the membranes prepared using PVC as the base polymer, variations were observed between the membrane containing only polymer and the one including 50% TOMATS, with contact angle values of 75.94° and 45.95°, respectively. For membranes prepared with a plasticizer, contact angle values ranged from 31.27° to 56.95°, with the lowest value for the membrane prepared with DOS and the highest for the one containing DBS.

BTS performed as one of the plasticizers that enhanced membrane hydrophilicity for both CTA- and PVC-based PIMs and therefore was considered as a suitable plasticizer for PIMs with both polymers mentioned above.

#### 3.1.2. Scanning Electron Microscopy (SEM) Imaging

**Figure 1:**
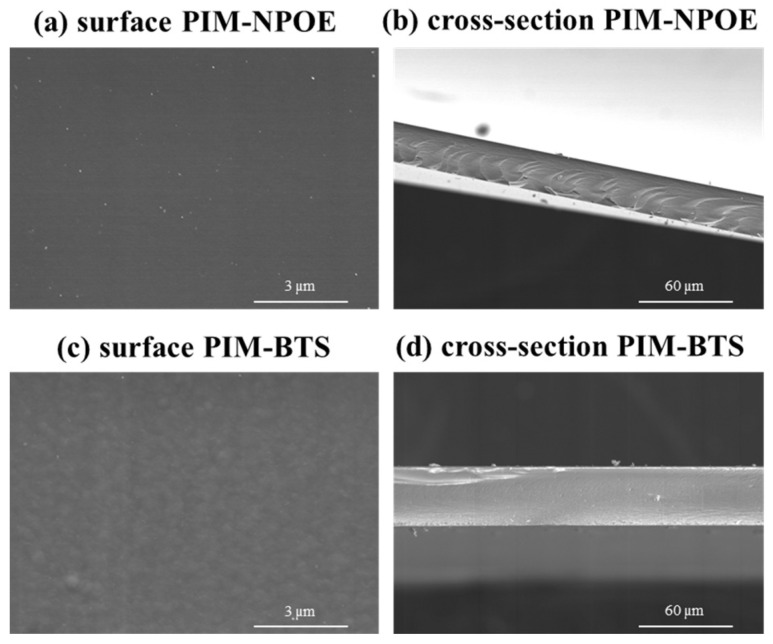
SEM images of CTA-based PIMs (50% CTA, 30% TOMATS, and 20% plasticizer (**a**,**b**) NPOE or (**c**,**d**) BTS).

**Figure 2:**
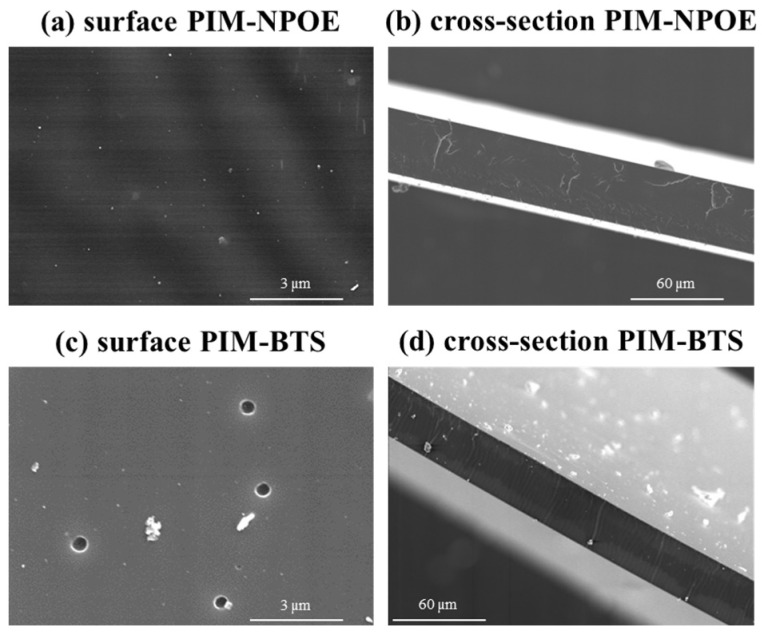
SEM images of PVC-based PIMs (50% CTA, 30% TOMATS, and 20% plasticizer (**a**,**b**) NPOE or (**c**,**d**) BTS).

The impact of the plasticizers NPOE and BTS on the microstructure of the membrane containing CTA (Figure 1) or PVC (Figure 2) as base polymers was studied. NPOE was selected as it is widely used in PIM fabrication and, in addition, it improved the Hg(II) TE [16], and BTS was selected in this study as a novel and promising plasticizer for manufacturing PIMs.

PIMs are usually described as homogeneous membranes, with no apparent porosity. In the case of CTA-based membranes, both NPOE and BTS produced membranes with similar characteristics, i.e., a dense structure and a smooth surface (Figure 1).

In the case of PVC-based membranes, no differences were observed when NPOE was used as a plasticizer (Figure 2a,b) compared to the CTA-based PIMs. In the case of BTS, the membranes featured indentations at the nano-sized scale which were scattered on their surface (Figure 2c). These indentations were formed due to solid–liquid separation because of solvent evaporation, as described in [25,26]. As expected, the cross-section image (Figure 2d) revealed that the membrane was microscopically dense, thus exhibiting the non-porous nature typical of PIMs.

#### 3.1.3. Mechanical Properties

Typically, PIMs exhibit favourable mechanical properties, including good resistance and flexibility. The mechanical characteristics of PIMs are evidently influenced by their composition [24]. As previously highlighted, a base polymer contributes to a PIM’s mechanical strength, while a plasticizer provides elasticity and flexibility to the membrane [23,27]. For this reason, it is to be expected that the properties may vary depending on the nature of the PIM components. Therefore, the effect of the polymer and the plasticizer on the mechanical properties of PIMs was evaluated for both CTA- and PVC-based membranes.

**Figure 3:**
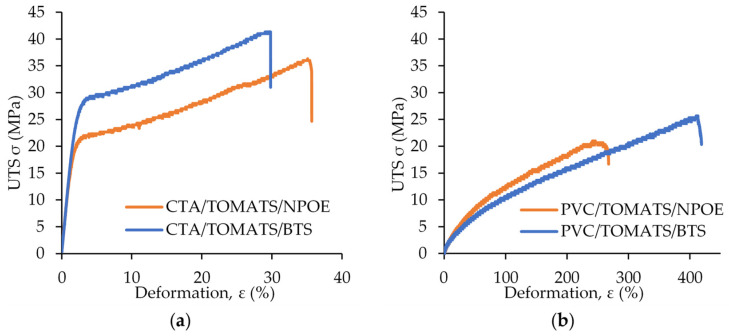
Ultimate tensile strength (UTS) for PIMs made of (**a**) CTA and (**b**) PVC and the corresponding plasticizer.

Tensile tests were conducted to assess the mechanical properties of the membranes. Figure 3 shows the UTS results for PIMs made of both CTA (Figure 3a) and PVC (Figure 3b) and for the plasticizers NPOE and DBS. It can be observed that, in general, the elastic modulus values of the CTA-based PIMs were much greater than those of the PVC-based membranes and that the maximum deformation values were much larger for the PVC-based membranes. These results indicate a higher mechanical resistance of the CTA-based PIMs compared to the PVC-based PIMs studied.

The behaviour of both the CTA- and PVC-based PIMs was consistent with that of the corresponding PIMs without plasticizer. In the absence of plasticizer, the CTA-based PIMs exhibited a higher stiffness than their PVC counterparts, which was reflected by the CTA’s higher Young’s modulus compared to PVC. In this context, it could be expected that the incorporation of plasticizers with different properties, such as NPOE and BTS, may have exerted a more pronounced influence on the PVC-based PIMs because of their lower stiffness compared to their CTA counterparts. This is in line with the obtained results, i.e., the use of BTS as a plasticizer in the PVC membranes drastically affected their elasticity, since the ɛmax (%) increased from 244 to 393 when compared with PIMs containing NPOE instead. However, in the case of CTA-based PIMs, the difference between the plasticizers was not significant. 

In terms of polymer science, the primary objective of adding a plasticizer to a polymer matrix is to enhance the material’s processability, characterized by improved deformability. This enhancement stems from the intercalation of a plasticizer within polymer chains, thus facilitating macromolecular mobility. For PVC, incorporating a long-chain plasticizer could augment this effect. The longer C–C chain of BTS compared to NPOE could be considered as responsible for the observed increase in elongation at break for the BTS-based PIMs. The elongation at break of the formulation plasticized by BTS was 1.6 times higher than that of the formulation plasticized by NPOE.

Regarding the CTA-based PIMs, the higher stiffness of CTA did not yield a distinct impact from the addition of plasticizers with varying C–C lengths. Consequently, the CTA-based PIMs with the different plasticizers exhibited closer values of deformability.

Table 3 presents the mechanical characteristics of the PIMs with all the plasticizers studied, including the average values of maximum deformation, maximum resistance, and elastic modulus. For comparison purposes, the same mechanical characteristics of the CTA and PVC are also presented.

The values of the UTS of the pure polymer membranes obtained in the current study were 83.0 ± 12.5 MPa for CTA and 57.0 ± 6.2, for PVC. Moreover, the inclusion of the PIM liquid phase (i.e., extractant and plasticizer) decreased the UTS and increased the value of elongation at break for all PIMs. Similar results were also reported by Nasser et al. [22] and Sellami et al. [23]. In the former study, a PIM composed of CTA and poly(butylene adipate-co-terephthalate) (PBAT) as the base polymers and Aliquat 336 as the carrier was employed for the selective transport of Cr(VI). The Young’s modulus of the pure CTA membrane was 3.0 ± 0.1 GPa. Moreover, it was reported that the PIMs prepared with Aliquat 336 exhibited higher deformability than the pure polymer membrane. The latter study evaluated a PIM composed of CTA, Aliquat 336, and DOS for the removal and recovery of Ag(CN)_2_^−^. In this case, the study found that the tensile strength of the CTA blank membrane was higher than that of the CTA-based PIM due to the inclusion of the carrier in the matrix causing a decrease in the Young’s modulus. Furthermore, it was reported that both pure CTA membranes and CTA-based PIMs had better mechanical properties than their PVC counterparts.

#### 3.1.4. Thermal Properties

To assess the thermal stability of the membranes, thermogravimetric analysis (TGA) was performed. While TGA primarily quantifies the mass change in a sample as a function of temperature and time in a controlled atmosphere [28,29,30], it offers valuable information regarding the thermal degradation characteristics and potential interactions among membrane components.

**Figure 4:**
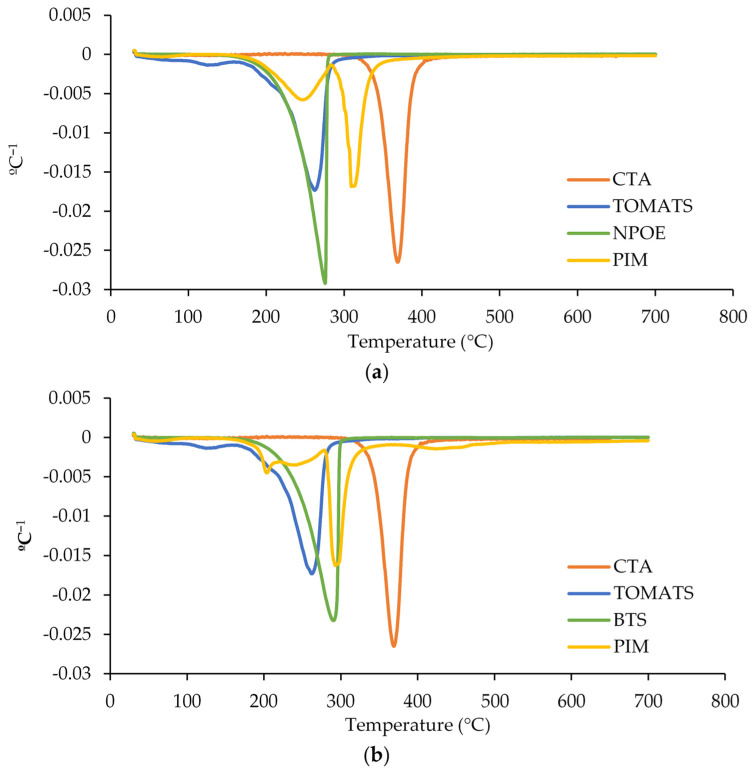
The dTGA profiles of CTA-based PIMs containing (**a**) NPOE and (**b**) BTS and their raw components.

**Figure 5:**
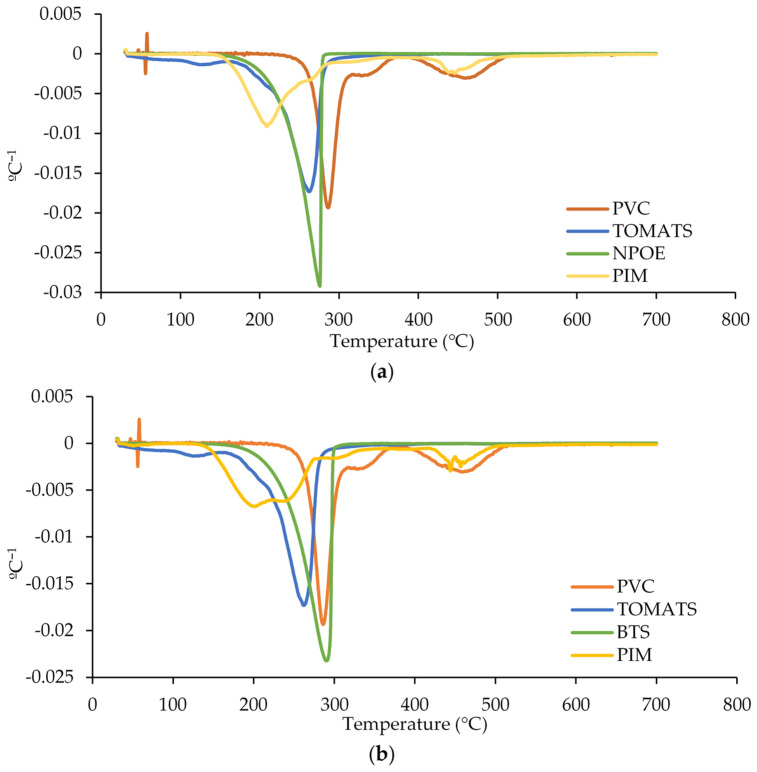
The dTGA profiles of PVC-based PIMs containing (**a**) NPOE and (**b**) BTS and their raw components.

The thermal properties of the pure components (CTA, PVC, TOMATS, NPOE, and BTS) as well as the corresponding membranes (see Figure 4 for CTA and Figure 5 for PVC) were investigated. Regarding the pure components, the degradation of pure CTA occurred in one step (370 °C) (Figure 4), while that of PVC, in two steps (285 °C and 460 °C) (Figure 5). These results agree with the study of Sedkaoui et al. [31], which described that the degradation step of CTA was attributed to the elimination of the acetate substituents and the formation of a pyranose ring in the molecule. In the case of PVC, the first step was attributed to the dehydrochlorination of the PVC and the formation of polyene sequences while the second one corresponded to the thermal decomposition of the dechlorinated PVC consisting mainly of conjugated double bonds. The other components of the PIMs, i.e., the carrier TOMATS and the plasticizers NPOE and BTS, had one thermal degradation step each, being approximately at 260, 275, and 290 °C, respectively.

Once these components were mixed to form the PIM, the degradation of the membranes took place at lower temperatures than those of the pure polymer, evidencing that the PIM was less thermally stable than the corresponding pure polymer (as shown in [32]). In the case of CTA membranes, the degradation at a higher temperature corresponded to the degradation of the polymer (at 311 °C instead of 370 °C) while the second was attributed to the degradation of TOMATS and the plasticizer (see Figure 4a for NPOE and Figure 4b for BTS).

Similar results were found for the PVC membranes, as shown in Figure 5a for NPOE and Figure 5b for BTS. In this case, it was not possible to distinguish between the degradation of the polymer and the other components of the membrane, since the degradation temperature of the pure PVC was close to those of both the TOMATS and plasticizer.

### 3.2. Effect of PIM Composition on Transport Performance of Hg(II) as a Case Study

**Figure 6:**
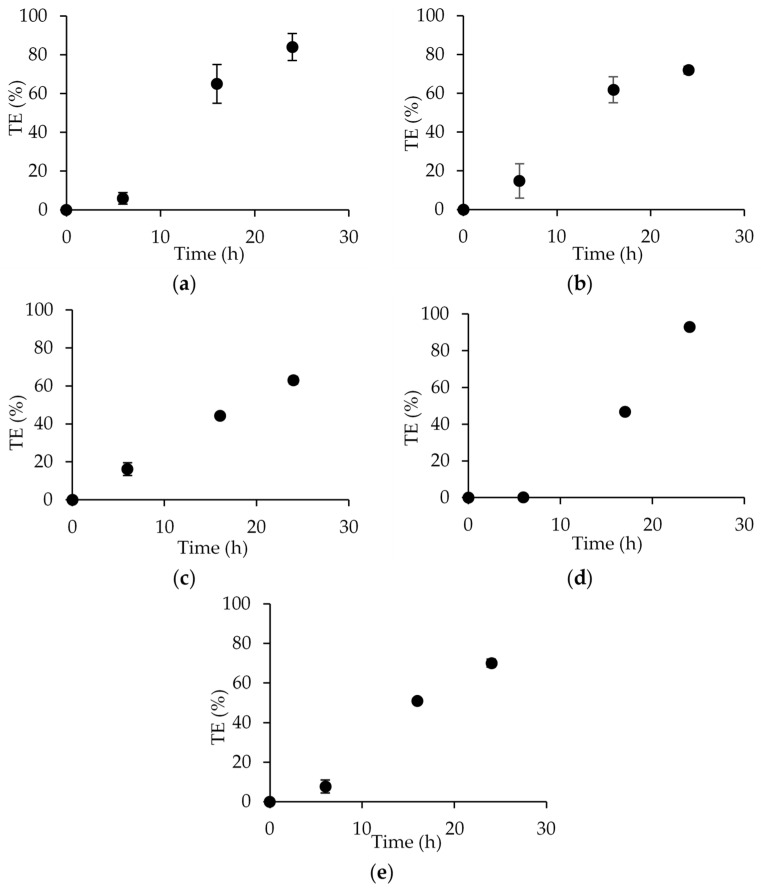
Hg(II) transport vs. time using PIMs made of 50% CTA, 30% TOMATS, and 20% plasticizer. The plasticizers used were (**a**) NPOE, (**b**) BBPA, (**c**) DOS, (**d**) DBS, and (**e**) BTS.

To assess the transport performance of membranes with various plasticizers, we selected Hg(II) as the model target ion for investigation. This choice was based on our previous findings where a PIM made of CTA, TOMATS, and NPOE was very effective in the removal of this toxic metal ion from natural waters [16]. Therefore, PIMs made of CTA and the plasticizers NPOE, BBPA, DOS, DBS, and BTS were used in the transport experiments. Hg(II) in the stripping compartment of the transport system was measured at different times for each membrane and the results are presented in Figure 6.

It was observed that all plasticizers facilitated the transport of Hg(II), with an increasing value of TE (%) over time. The PIM with DBS allowed for the quantitative transport of the metal ion in 24 h, while the other plasticizers exhibited TE values in the range of 63–84% for the same transport time. Moreover, the PIM with BTS showed a similar efficiency, with a TE value of 70% after 24 h of transport. 

Transport experiments (24 h) using PVC-based PIMs with either NPOE or BTS as the plasticizer were also conducted. In this case, the nature of the plasticizers had a significant impact on the transport of Hg(II). The NPOE membrane achieved a TE of only 18 ± 4%, while quantitative transport was accomplished for the BTS membrane (95 ± 7%). This result is consistent with the better plasticizing capacity of BTS observed for the PVC polymer in the mechanical characterization studies. BTS, with a long aliphatic chain, better separates the chains of the polymer than NPOE (which is considered more rigid due to its aromatic ring) and better facilitates the diffusion of the extracted species within the membrane.

## 4. Conclusions

In this work, it has been demonstrated that BTS can successfully be used as a plasticizer in PIMs containing TOMATS as the carrier and PVC or CTA as the base polymer. The measured properties of the PIMs containing BTS, such as hydrophilicity and mechanical properties, were in the range of those of the PIMs with the other plasticizers tested. In terms of Hg(II) transport efficiency, the utilization of BTS in PVC membranes significantly enhances membrane performance compared to the plasticizer NPOE. This improvement can be attributed to the capacity of long-chain aliphatic molecules to disperse the PVC network more effectively. Given BTS’s positive performance and cost-effectiveness, it emerges as a promising alternative to current plasticizers in PIM formulations.

## Figures and Tables

**Table 1:** Characteristics and chemical structure of the studied plasticizers.

Plasticizer	Chemical Structure	Viscosity (cP)	Dielectric Constant (ε_r_)	Density (g mL^−1^)	Ref.
2-Nitrophenyl octyl ether (NPOE)	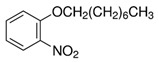	11.1	24	1.04	[18]
Diethylhexyl phthalate (DEHP)	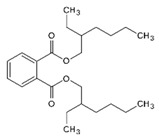	40.4	5.22	0.99	[18]
Bis(1-butylpentyl) adipate (BBPA)	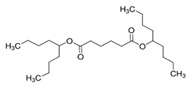	14	4	0.91	[7]
Dioctyl sebacate (DOS)	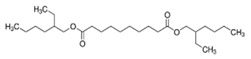	16.7	3.9	0.91	[1]
Dibutyl sebacate (DBS)	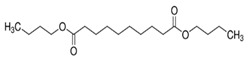	9.5	4.5	0.94	[18]
Butyl stearate (BTS)	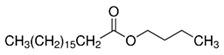	9.2 ^1^	3.1	0.86	[19]

^1^ Determined as part of the current study (at 20 °C).

**Table 2:** Contact angle (°) values of PIMs (50% polymer, 30% TOMATS, and 20% plasticizer) with different plasticizers.

Membrane Composition	Contact Angle (°) CTA-Based PIM	Contact Angle (°) PVC-Based PIM
100% polymer	62.62 ± 0.59	75.94 ± 1.47
50% polymer–50% TOMATS	49.27 ± 0.78	45.95 ± 3.91
NPOE	42.54 ± 3.22	41.92 ± 1.04
DEHP	41.50 ± 1.2	50.88 ± 3.35
BBPA	39.90 ± 2.01	43.83 ± 4.32
DOS	49.45 ± 1.33	31.27 ± 2.80
DBS	39.40 ± 2.27	56.95 ± 3.45
BTS	34.90 ± 4.27	47.11 ± 5.72

**Table 3:** Maximum deformation (ɛmax, %), ultimate tensile strength (σmax, MPa), and elastic modulus (E, MPa) of PIMs composed of 50% polymer, 30% TOMATS, and 20% plasticizer (*n* = 2–5).

PIM Composition	CTA-Based PIM			PVC-Based PIM		
	εmax (%)	σmax (MPa)	E (MPa)	εmax (%)	σmax (MPa)	E (MPa)
100% polymer	n.d.	83.0 ± 12.5	n.d.	n.d.	57.0 ± 6.2	n.d.
NPOE	34.4 ± 5.2	35.5 ± 0.8	1828.8 ± 649.7	243.9 ± 3.5	19.5 ± 2.1	266.7 ± 76.2
DEHP	n.d.	24.3 ± 3.5	n.d.	300.4 ± 26.2	22.8 ± 4.5	395.4 ± 9.4
BBPA	20.3 ± 10.4	32.0 ± 3.7	1348.0 ± 98.3	269.1 ± 30.0	16.8 ± 2.6	397.6 ± 14.1
DOS	20.1 ± 11.2	29.4 ± 5.3	1357.2 ± 135.2	n.d.	3.9 ± 0.3	n.d.
DBS	24.5 ± 9.7	29.2 ± 8.6	1335.5 ± 306.4	127.5 ± 2.9	7.3 ± 0.9	274.8 ± 131.6
BTS	25.4 ± 8.8	37.3 ± 10.6	1543.8 ± 250.9	393.0 ± 24.4	26.8 ± 1.7	310.5 ± 45.6

(n.d. = not determined).

## Data Availability

Data are contained within the article.
